# A standard descriptor for fixed-target serial crystallography

**DOI:** 10.1107/S2059798323005429

**Published:** 2023-07-18

**Authors:** Robin L. Owen, Daniele de Sanctis, Arwen R. Pearson, John H. Beale

**Affiliations:** a Diamond Light Source, Harwell Science and Innovation Campus, Didcot OX11 0DE, United Kingdom; b ESRF – The European Synchrotron, 71 Avenue des Martyrs, 38000 Grenoble, France; c Institute for Nanostructure and Solid State Physics, Hamburg Centre for Ultrafast Imaging, Universität Hamburg, HARBOR, Luruper Chaussee 149, 22761 Hamburg, Germany; d Paul Scherrer Institute, Forschungsstrasse 111, 5232 Villigen, Switzerland; University of Oxford, United Kingdom

**Keywords:** fixed targets, serial crystallography, standardization

## Abstract

Fixed-target crystallography has become a widely used approach for serial crystallography at both synchrotron and X-ray free-electron laser (XFEL) sources. A plethora of fixed targets have been developed at different facilities and by various manufacturers, with different characteristics and dimensions and with little or no emphasis on standardization. These many fixed targets have good reasons for their design, shapes, fabrication materials and the presence or absence of apertures and fiducials, reflecting the diversity of serial experiments. Given this, it would be a Sisyphean task to design and manufacture a new standard fixed target that would satisfy all possible experimental configurations. Therefore, a simple standardized descriptor to fully describe fixed targets is proposed rather than a standardized device. This descriptor is a dictionary that could be read by fixed-target beamline software and straightforwardly allow data collection from fixed targets new to that beamline. The descriptor would therefore allow a much easier exchange of fixed targets between sources and facilitate the uptake of new fixed targets, benefiting beamlines, users and manufacturers. This descriptor was first presented at, and was developed following, a meeting of representatives from multiple synchrotron and XFEL sources in Hamburg in January 2023.

## Abstract 2

A descriptor with a standard format and parameters is introduced for the large-area fixed targets used in serial crystallography. It is proposed that any fixed target used and developed by different facilities or groups could have its own version of this descriptor, facilitating their wider use and exchange.

## 1. Introduction

Fixed targets have become a popular sample-delivery approach in serial femtosecond crystallography (SFX) and serial synchrotron crystallography (SSX) at X-ray free-electron laser (XFEL) and synchrotron light sources, respectively. As they are relatively simple to design, adapt and adopt, a wide variety of fixed-target devices have been developed and used successfully at a large number of sources (Roedig *et al.*, 2016 ▸, 2017 ▸; Oghbaey *et al.*, 2016 ▸; Hunter *et al.*, 2014 ▸; Doak *et al.*, 2018 ▸; Ren *et al.*, 2018 ▸; Lee *et al.*, 2019 ▸; Horrell *et al.*, 2021 ▸; Sherrell *et al.*, 2022 ▸; Mehrabi *et al.*, 2020 ▸). While users and facility staff alike are now in the enviable position where ‘off-the-shelf’ products are within reach, the separate development of so many fixed targets for serial sample delivery, each often optimized for a particular experiment, has perhaps now become more challenging than enhancing. Even the nomenclature adds to the confusion, with terms such as fixed targets, chips, thin-film sandwiches, meshes and solid supports all being used interchangeably. Given the range of possible solutions in the literature and their continual development, it is never completely clear which targets are supported and how, or whether a new fixed target could be accommodated at a particular beamline. Some degree of standardization, therefore, would benefit all.

However, standardization across a diverse landscape of solutions is a thankless task and not one seen as particularly enjoyable. We feel however that fixed target serial crystallography is evolving to a point where standardization of key components will greatly facilitate future creativity. With this in mind, a workshop was organised in January 2023 and kindly hosted by the European XFEL as part of their user meeting to address this challenge.

The goal of the workshop was to identify potential areas of standardization between fixed targets and to work towards best practices in data collection and recording. The two principal concerns were to increase inter-facility fixed-target exchange, whilst not stifling innovation, and also not to impose restrictions on the site-specific hardware that currently enables fixed-target serial crystallography at existing beamlines. It rapidly became apparent that it would be extremely difficult to reach broad agreement on a single, or indeed a small number of, economical fixed-target designs that would satisfy all current serial experiments. We instead propose a simple standard descriptor that can be used to describe all aspects of any fixed target relevant to motion during data collection, with the aim that if this is provided to the beamline software then data collection will be straightforward to implement. The descriptor therefore provides all of the parameters required for the alignment, and subsequent movement, of a fixed target through the X-ray beam.

## 2. Fixed targets

### 2.1. Scope of description

**Figure 1:**
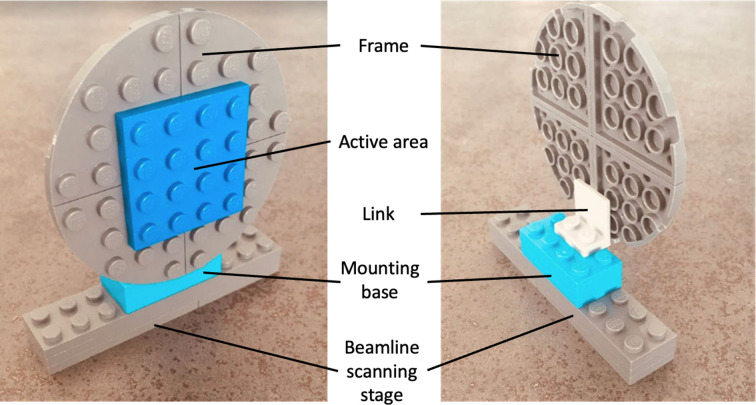
A schematic model of a fixed target highlighting the principal components. Design and construction courtesy of Gabriel R. de Sanctis.

Serial crystallography fixed targets are typically comprised of four component parts: a mounting base, a connecting link, a frame and an active area (Fig. 1 ▸). The active area is where the crystals are mounted, and the frame then holds this in place. The mounting base is the interface to the beamline scanning stages and the connecting link joins the frame to the base. We specify the link as a separate entity since the distance between the mounting base and the target is of relevance for the positioning of the chip active area and can often vary between different fixed-target solutions and with the geometry of the scanning stage. We note that this specific aspect is an area for future standardization. In the vast majority of cases, a single mounting base, connecting link and frame are used to accommodate a range of different fixed targets (active-area configurations) at a particular endstation. We have therefore restricted the scope of our standard description to the active area and use the following definition of a serial crystallo­graphy fixed target to which our descriptor can be applied:

Serial crystallography fixed targets are solid supports that can hold multiple crystals in either defined or random locations, typically in a single plane. Once aligned, the target is moved through the X-ray beam with data collected at a set of predefined positions.

Some sort of alignment strategy is essential for fixed-target serial crystallography to ensure the correct placement of the active area in *x*, *y* and *z* with respect to the X-ray beam during data collection.^1^ Combined motion of the translation stages holding the fixed target allows defined positions of the active area to be moved into the X-ray beam path and ensures that the crystal-to-detector distance remains constant. A consistent *z* placement also ensures that the spatial overlap of any laser or droplet ejection used for reaction initiation in a time-resolved experiment remains the same over the entire active area. Fixed-target data-collection modes can be broadly grouped into two types.

(i) Directed raster: a raster grid is drawn over a chip where crystals are randomly distributed on the active area. The stages then move sequentially through this generated grid. X-rays could theoretically hit anywhere on the target.
(ii) Aperture aligned: crystals are loaded into defined cavities on the active area of the chip. The stages sequentially move each aperture into alignment with the X-ray beam. X-rays will only hit/pass through the apertures.

We propose a standard dictionary that can describe both types of fixed-target active area. Each chip type will have its own unique list of definitions, but these are set into a common file format that can be read by either generic or specific facility software. The descriptor presented here aims to fully describe regular arrays, allowing alignment and subsequent data collection in a concise, human-readable format. *yaml* (https://yaml.org) provides a simple means of achieving this and can easily be generated from a text editor or from the command line and parsed by beamline data-acquisition software. While the descriptor aims to provide all of the parameters required for motion control, it does not include the information required to design a suitable frame since the beamline software and motion control are agnostic to this.

We note that fixed-target data collection may also follow an irregular path to, for example, hit only previously identified or pre-characterized samples on any type of fixed target. The standard descriptor could facilitate data collection from such a ‘mapped’ active area if the mapping were performed offline and fiducial or feature-based alignment of the sample holder is required prior to data collection. The descriptor does not contain either a list of all points on an active area that should be visited or a preferred order of points for data collection.

### 2.2. Worked example

**Figure 2:**
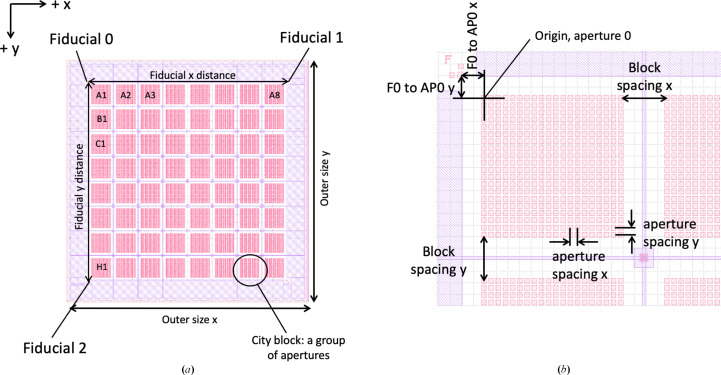
Schematic of a fixed target with the parameters labelled. (*a*) shows the entire fixed target, which comprises 64 city blocks with outer dimensions of approximately 30 × 30 mm. Each city block contains 400 apertures. There is a fiducial marker for alignment at each corner of the fixed target. (*b*) highlights a subregion of the fixed target showing a single city block. Distance values for this and other fixed targets are given in Table 2 ▸.

Fig. 2 ▸ shows a schematic of a fixed target developed and used at Diamond Light Source that will be used as an example to describe each of the dictionary parameters. The description uses the following assumptions and conventions.

(i) The apertures (or active area for a directed raster) lie on a common plane.
(ii) The origin is at the top left of the target, with *x* in the horizontal and in *y* in the vertical, with positive *x* and *y* motion describing motion away from the origin across the target (Fig. 2 ▸).
(iii) External dimensions are not required to define the motion during serial data collection and so are not included in the description.
(iv) Distances are from centre of feature to centre of feature, not from edge to edge.
(v) Distances are in millimetres.

While the assumption is made that all of the apertures lie on a common plane, an offset of the fiducials in and out of this plane (*i.e.* in *z*) could be incorporated into the dictionary in the case where, for example, the fiducials lie out of the focal plane where the samples are held. A list, and descriptions, of the parameters included in the descriptor is given in Table 1 ▸.

### 2.3. Notes on fixed-target parameters and descriptions

The above assumes that in the case of apertured chips all apertures lie directly above/below each other when moving between rows. If rows are offset in, for example, a honeycomb or other more esoteric pattern then the ‘geometry type’ allows the future incorporation of this information. However, we are unaware of any current or planned fixed targets that do not utilize a square array and so we do not expand further on this here.

If a value/field is missing then the value is assumed to be zero (with the exception of geometry type, which defaults to square). For example, if the corner apertures in the active area are used for alignment instead of dedicated fiducial features, then all quantities relating to the fiducials can be omitted and only the city block and aperture information is required for a full description.

**Figure 3:**
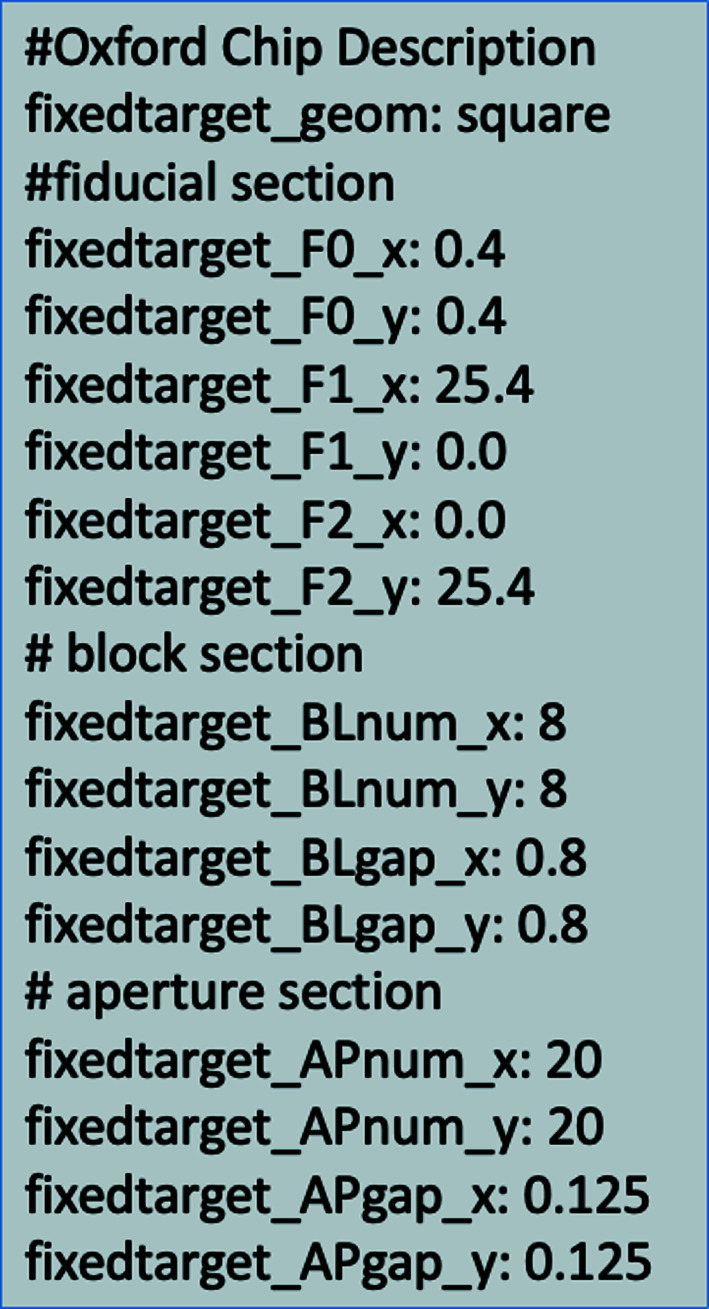
Complete standard description of the fixed target shown in Fig. 2 ▸.

Fig. 3 ▸ shows a complete description of the fixed target shown in Fig. 2 ▸ in the suggested *yaml* format. Table 2 ▸ expands this to give the parameters for complete descriptions of a number of fixed targets in common use. It is important to note that if pasted into a *yaml* file then the contents should be correctly formatted to the colon space delimited format. Correctly formatted *yaml* files for each of the fixed targets described in Table 2 ▸ are available as supporting information.

## 3. Conclusions

We suggest that a standard description of this type should be provided with all fixed targets and, similarly, descriptions of this type should be readable by all beamlines accommodating fixed-target serial data collection. This will enable a user’s ‘favourite’ fixed target to be used at multiple beamlines/sources. We hope that it will also allow commercially available fixed targets to be easily used at multiple beamlines and will facilitate switching between fixed-target formats as and when the experiment changes within a beamtime.

Further aspects of fixed-target serial crystallography also provide opportunities for future standardization. For example, agreement on the connecting link between the fixed-target and beamline scanning stages will be essential if automated sample exchange is to evolve beyond bespoke one-off solutions. Equally advantageous for all serial experiments will be a standardized recording of the metadata associated with each data collection. Standardization of these and other aspects of fixed-target serial crystallography will allow future work and creativity to focus on new functionality rather than on making existing experiments work. We hope that this standard descriptor of the fixed targets used for serial data collection will provide a first step in inter-source standardization in this constantly developing field.

## Supplementary Material

Standard descriptor of HARE chip (.yml file). DOI: 10.1107/S2059798323005429/gm5097sup1.txt

Standard descriptor of SOS chip (.yml file). DOI: 10.1107/S2059798323005429/gm5097sup2.txt

Standard descriptor of MISP chip (.yml file). DOI: 10.1107/S2059798323005429/gm5097sup3.txt

Standard descriptor of Oxford chip (.yml file). DOI: 10.1107/S2059798323005429/gm5097sup4.txt

## Figures and Tables

**Table 1:** Parameters included in the standard description and their definitions

Name	Key	Description
Geometry type		
Square, hex	fixedtarget_geom	Geometry type. If absent, square is assumed.
Fiducials		
Fiducial 0 *x* position	fixedtarget_F0_x	Distance from fiducial 0 to aperture 0 in *x*
Fiducial 0 *y* position	fixedtarget_F0_y	Distance from fiducial 0 to aperture 0 in *y*
Fiducial 0 *z* position	fixedtarget_F0_z	Distance from fiducial 0 to aperture 0 in *z*
Fiducial 1 *x* position	fixedtarget_F1_x	Distance from fiducial 0 to fiducial 1 in *x*
Fiducial 1 *y* position	fixedtarget_F1_y	Distance from fiducial 0 to fiducial 1 in *y*
Fiducial 1 *z* position	fixedtarget_F1_z	Distance from fiducial 0 to fiducial 1 in *z*
Fiducial 2 *x* position	fixedtarget_F2_x	Distance from fiducial 0 to fiducial 2 in *x*
Fiducial 2 *y* position	fixedtarget_F2_y	Distance from fiducial 0 to fiducial 2 in *y*
Fiducial 2 *z* position	fixedtarget_F2_z	Distance from fiducial 0 to fiducial 2 in *z*
City blocks		
Number in *x*	fixedtarget_BLnum_x	Number of city blocks in *x*
Number in *y*	fixedtarget_BLnum_y	Number of city blocks in *y*
Spacing in *x*	fixedtarget_BLgap_x	Gap between city blocks in *x*. Distance from the last aperture in block *n* to the first aperture in block (*n* + 1).
Spacing in *y*	fixedtarget_BLgap_y	Gap between city blocks in *y*
Apertures		
Number in *x*	fixedtarget_APnum_x	Number of apertures in *x* in each block
Number in *y*	fixedtarget_APnum_y	Number of apertures in *y* in each block
Spacing in *x*	fixedtarget_APgap_x	Distance between adjacent apertures in *x*
Spacing in *y*	fixedtarget_APgap_y	Distance between adjacent apertures in *y*

**Table 2:** Standard description for three aperture-aligned fixed targets in common use, Oxford (see, for example, Horrell *et al.*, 2021 ▸), HARE (Mehrabi *et al.*, 2020 ▸) and the MIcro-Structured Polymer (MISP) chip from PSI (unpublished work), and a directed raster, the Sheet-On-Sheet (SOS) chip from Heidelberg (Doak *et al.*, 2018 ▸) *yaml* files for each of these types of fixed target are included as supporting information.

Chip name	Oxford	HARE	MISP chip	SOS chip
fixedtarget_F0_x	0.4	0	1.78	0
fixedtarget_F0_y	0.4	0	1.78	0
fixedtarget_F1_x	25.4	25.2	23	25
fixedtarget_F1_y	0	0	0	0
fixedtarget_F2_x	0	0	0	0
fixedtarget_F2_y	25.4	25.2	23	25
fixedtarget_BLnum_x	8	6	1	1
fixedtarget_BLnum_y	8	6	1	1
fixedtarget_BLgap_x	0.8	0.9	0	0
fixedtarget_BLgap_y	0.8	0.9	0	0
fixedtarget_APnum_x	20	24	162	<user-defined>
fixedtarget_APnum_y	20	24	162	<user-defined>
fixedtarget_APgap_x	0.125	0.150	0.120	<user-defined>
fixedtarget_APgap_y	0.125	0.150	0.120	<user-defined>

## Footnotes

1 We use here the convention that *x* is in the horizontal direction, *y* is vertical and *z* is horizontal in the beam direction.
